# Low Rates of Lateral Gene Transfer among Metabolic Genes Define the Evolving Biogeochemical Niches of Archaea through Deep Time

**DOI:** 10.1155/2012/843539

**Published:** 2012-11-22

**Authors:** Carrine E. Blank

**Affiliations:** Department of Geosciences, University of Montana, 32 Campus Drive no. 1296, Missoula, MT 59812-1296, USA

## Abstract

Phylogenomic analyses of archaeal genome sequences are providing windows into the group's evolutionary past, even though most archaeal taxa lack a conventional fossil record. Here, phylogenetic analyses were performed using key metabolic genes that define the metabolic niche of microorganisms. Such genes are generally considered to have undergone high rates of lateral gene transfer. Many gene sequences formed clades that were identical, or similar, to the tree constructed using large numbers of genes from the stable core of the genome. Surprisingly, such lateral transfer events were readily identified and quantifiable, occurring only a relatively small number of times in the archaeal domain of life. By placing gene acquisition events into a temporal framework, the rates by which new metabolic genes were acquired can be quantified. The highest lateral transfer rates were among cytochrome oxidase genes that use oxygen as a terminal electron acceptor (with a total of 12–14 lateral transfer events, or 3.4–4.0 events per billion years, across the entire archaeal domain). Genes involved in sulfur or nitrogen metabolism had much lower rates, on the order of one lateral transfer event per billion years. This suggests that lateral transfer rates of key metabolic proteins are rare and not rampant.

## 1. Introduction

Although there are still active debates over the extent of lateral gene transfer (LGT) in prokaryotic genomes [1], phylogenomic analyses using large sets of slowly evolving universally present genes have been producing an increasingly clearer picture of the core evolutionary signal in prokaryotic genomes, particularly for the archaeal domain of life [2, 3]. The actual rates of lateral gene transfer (in terms of number of events per billion years) are largely unknown, yet there is a growing recognition that lateral transfer among some sets of genes, particularly metabolic genes, appears to be higher than in other sets of genes [4–7]. Such lateral transfer events have no doubt led to the acquisition of new traits that help to redefine the metabolic niche of microorganisms, and these events have therefore had a profound influence on the evolving biogeochemical cycles on the Earth [8, 9]. Previous work on the archaeal domain of life has suggested an emerging evolutionary conservation between the habitat preference and metabolic traits [10]. These niches have been changing through deep time as biogeochemical cycles gradually became more complex, particularly once oxygen became available in the biosphere. 

This work is set about explicitly quantifying the rate of lateral gene acquisition events amongst key metabolic genes in the archaeal domain of life, using a phylogenetic approach. Phylogenetic trees of metabolic genes were constructed, and clades that were congruent with the slowly evolving core phylogenomic signal were identified in order to quantify the number of lateral gene transfer events (gains in new metabolic traits) for each gene. Inferred node ages were then used to identify when, in deep time, new metabolic traits could have been acquired via lateral gene transfer. This study shows that lateral transfer rates were highest among the genes using oxygen as a terminal electron acceptor, and lowest amongst genes involved in redox reactions between sulfur and sulfide. Nevertheless, the overall rate of metabolic gene acquisition in the archaeal domain appears to have been low—with cytochrome oxidase having the highest average rate of 3.4–4.0 events per billion years.

## 2. Methods

### 2.1. Concatenated Genomic Phylogeny, Node Ages, and Ancestral State Reconstruction

The methods for calculating the genomic tree and converting the branch lengths to ages have been published in detail elsewhere [10, 11]. In brief, the backbone genomic tree topology for the archaeal domain of life was calculated with Bayesian and maximum parsimony methods using a concatenated genomic dataset containing 98 protein and 2 ribosomal RNA sequences. The genomic dataset contained 119 taxa from cultured representatives of the Crenarchaeota and Euryarchaeota. The dataset contained a diverse suite of protein and ribosomal RNA sequences that sampled the core functioning of the cell. Ribosomal RNA sequences were aligned using secondary structure in order to align homologous nucleotides [12]. Protein sequences were aligned using CLUSTALW [13] and hand edited in Se-Al [14], and character sets were defined such that only unambiguously aligned regions were used to construct the phylogenetic trees. Bayesian trees were calculated using MrBayes (v. 3.1.2 and 3.2.1; [15]) and maximum parsimony trees using PAUP* [16]. These trees formed the core set of branching relationships that defined the consensus genome tree.

**Figure 1:**
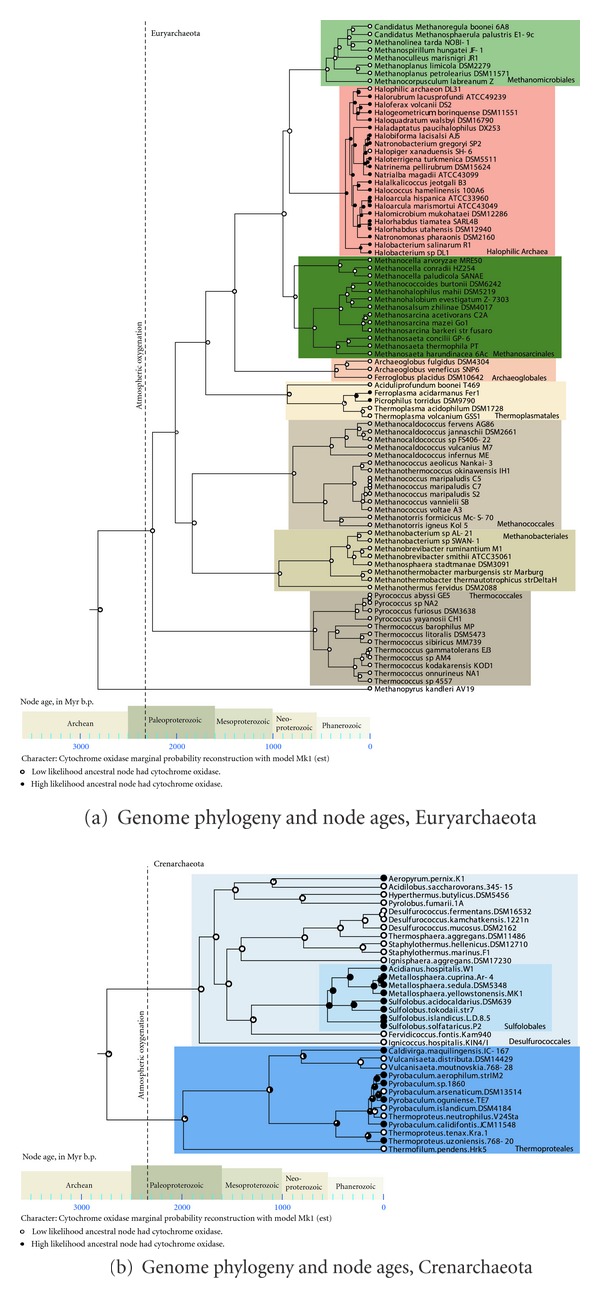
Genome phylogeny and node ages. Node ages (scale at bottom, in millions of years before present) were inferred using relaxed molecular clock method penalized likelihood. The presence of a cytochrome oxidase (subunit I or subunit I+III from either Heme-copper oxygen reductase family A1 or B) was coded in Mesquite as 0 = absence in the genome or 1 = presence in the genome. The maximum likelihood method (implementing the Mk1 model) was used to estimate the probability that each ancestral genome in the tree (each node) contained a cytochrome oxidase gene. A completely white circle indicates zero likelihood of a cytochrome oxidase gene; a completely black circle indicates a 100% likelihood, while intermediate shading of the pie indicates an intermediate likelihood. The corresponding geologic age of the nodes in the tree can be determined using the age diagram at the bottom. The archaeal tree was split into two figures, (a) one showing the Euryarchaeota and the other (b) showing the Crenarchaeota, for ease of viewing. Major archaeal clades are indicated using colored boxes, with the appropriate family names shown.

In order to scale branch lengths to time in Figure 1, branch lengths for the tree were estimated with the maximum likelihood method in PAUP*, using the large and small ribosomal RNA sequences, were the maximum likelihood parameters (pinvar, gamma distribution, nucleotide frequencies, and the substitution rate matrix) were estimated from the data using the GTR + I + Γ model. The genome tree (calculated using all 100 protein and rRNA sequences) was used as a backbone constraint tree. Branch lengths were then converted to geologic ages using the penalized likelihood method in r8s [17, 18] using multiple age constraints. The maximum and minimum ages of the root of the archaeal domain were set at 3.8 Ga and 2.7 Ga, and the minimum age of the Euryarchaeota was 2.7 Ga [10]. Oxygen age constraints were used to constrain the maximum age (2.32 Ga) of the Thermoplasmatales, Sulfolobales, Thermoproteales, *Pyrobaculum-Thermoproteus*, and the Halobacteriales [19]. The oxygen age constraint of 2.32 Ga (the time by which free oxygen appeared in the atmosphere) was applied for archaeal clades that have the ability to use oxygen as a terminal electron acceptor, likely had an ancestor that metabolized oxygen, had an ancestor with a likely gene for metabolizing oxygen, and lived in an environment where cyanobacteria (a local source of oxygen) could not have been present. Chitin maximum age constraints (1.0 Ga, when chitin first appeared in the biosphere) were set for the Halobacteriales and Thermococcales, and the lignin maximum age constraints (475 Ma, when lignin from land plants first appeared) were set for the *Sulfolobus solfataricus-Sulfolobus islandicus* clade [19]. Chitin maximum age constraints were applied for clades that currently have the ability to metabolize chitin and that likely had an ancestor with the ability to metabolize chitin. Lignin maximum age constraints were applied for clades that currently have the ability to metabolize phenolic compounds derived from lignin and that likely had an ancestor with the ability to metabolize phenolic compounds derived from lignin. The presence of cytochrome oxidase subunit I (or subunit I+III) was coded in Mesquite v. 2.75 [20] such that 0 = absence of cytochrome oxidase in the genome and 1 = presence. Ancestral state reconstruction was performed in Mesquite using the likelihood method MK1 model, using the tree where branch lengths were scaled according to geologic time (Figure 1). Such an analysis shows the estimated geologic time where archaeal clades have acquired the ability to metabolize oxygen using a cytochrome oxidase gene. Such nodes are strong candidates for acquisition of metabolic genes via the process of lateral gene transfer.

### 2.2. Functional Gene Phylogenies and Topology Tests

The metabolic genes chosen for this study were a subset of those that play fundamental roles in the biogeochemical cycling of oxygen, sulfur, nitrogen, and carbon through the biosphere. Phylogenetic trees of metabolic genes were constructed by obtaining protein sequences from microbial genome sequences in GenBank using TBLASTN against the NCBI Chromosome and WGS databases. Database searches began using metabolic genes with demonstrated biochemical activity (from either bacteria or archaea, where available) and homologs were obtained from GenBank using TBLASTN against the nonredundant database. Orthology assessment was made using a pairwise orthology approach [21]. Genes sequences were chosen if they showed BLAST scores that were significantly better than the highest scores to other annotated orthologs or genes with a different demonstrated biochemical activity. Next, phylogenetic trees of metabolic protein alignments were carried out using Clustal W, hand edited, and exported as nexus files, and phylogenetic trees were constructed using the maximum parsimony method in PAUP* v. 4.0b10 [16] and the Bayesian method in MrBayes v.3.1.2 and 3.2.1 [15]. Congruence testing of nitrogenase topologies was carried out using the approximately unbiased (AU) test in CONSEL [22], where site likelihoods were calculated with MrBayes. 

### 2.3. Quantifying Lateral Transfer Rates

**Figure 2:**
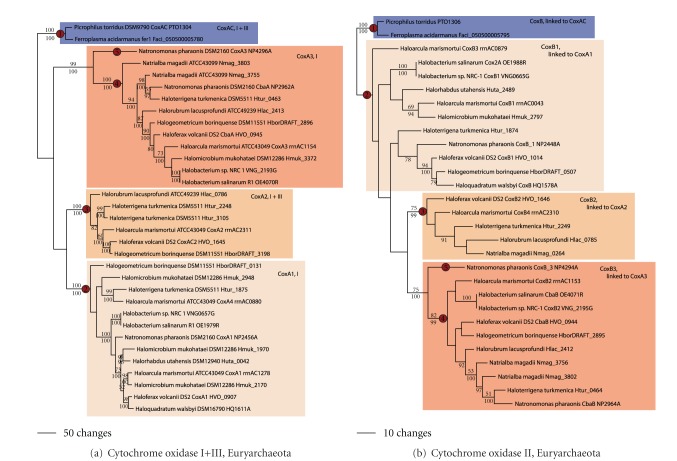
Cytochrome oxidase in the Euryarchaeota. Phylogenetic trees for (a) cytochrome oxidase subunit I and (b) cytochrome oxidase subunit II. Putative lateral transfer events are indicated using a filled red circle. The locus tag numbers are provided for taxa with genome sequences (accession numbers are provided for taxa without genome sequences). Trees shown have branch lengths and relationships constructed using the maximum parsimony method. Bootstrap values above 50% from the maximum parsimony method are shown above each branch, and branches without values had less than 50% support. Posterior probabilities from the Bayesian method are shown below each branch. Major archaeal clades are indicated using colored boxes, and potential lateral gene transfer events are identified at the base of the node for these clades with a filled red circle. Nodes discussed in the text were assigned sequential circled numbers.

Candidate lateral gene transfer events (in this study, the acquisition of novel metabolic genes) were first identified using ancestral state reconstruction. Potential lateral transfer events can be identified if a clade has an ancestor that likely lacked a particular trait and then later gained the trait higher up in tree (e.g., cytochrome oxidase subunit I in Figure 1). Next, transfer events were confirmed if phylogenetic analysis of the protein sequencing underlying the trait exhibited moderately or well-supported branching relationships (as determined by bootstrap analyses or posterior probabilities greater than 70%) that were inconsistent with the concatenated genomic phylogeny. Relationships within clades were also examined; clades with internal relationships consistent with the genome phylogeny, yet showing an alternative rooting (e.g., CoxA1 and CoxA3 in Figure 2(a)), were also considered to have likely undergone lateral gene transfer, possibly from a donor outside of the archaeal domain of life. Next, the position of each gene was examined in relation to other subunits in the enzyme, or in other enzymes in a pathway that could have been transferred in a single transfer event. Genomic positional linkages were noted so as to conservatively estimate lateral transfer rates. If two genes were located in an operon that coded for a multienzyme complex or proteins in the same metabolic pathway, they were likely acquired in a singular lateral transfer event.

The overall rates of lateral gene transfer for the archaeal domain of life were calculated by summing up the total number of inferred lateral transfer events (the gains of new metabolic genes involved in key biogeochemical cycles) over the estimated age of the archaeal domain of life. These inferred LGT acquisition rates are summed up for the entire domain of life, assuming that the domain is comprised of the 119 cultured archaeal taxa in the Crenarchaeota and Euryarchaeota that make up this study.

## 3. Results and Discussion

### 3.1. Genome Phylogeny, Ancestral State Reconstruction, and Relaxed Clock

Figure 1 is a phylogenetic tree constructed using a 100-gene concatenated dataset. The branch lengths are scaled to geologic time, using age constraints for the archaeal domain of life developed by Blank [11, 19], and the likelihood that the ancestral genomes have cytochrome oxidase is indicated on the nodes of the tree using ancestral state reconstruction. This approach inferred that the ancestor to the Halobacteriales likely contained cytochrome oxidase in its genome. It also infers a high likelihood that the ancestor to *Ferroplasma-Picrophilus* and the Sulfolobales contained cytochrome oxidase. There is a high likelihood that the ancestor to *Thermoproteus-Pyrobaculum* contained cytochrome oxidase, yet it is less likely that cytochrome oxidase traces further back in the Thermoproteales. This demonstrates how ancestral state reconstruction can be used to identify potential lateral gene transfer (LGT) events in terms of the acquisition of novel metabolic genes that help define the metabolic niche of an organism (in this case, aerobic respiration). Ultimately, however, to quantify the number of lateral transfer events, one must also closely examine the phylogenetic trees that underlie the trait in question in order to confirm the hypothesis that these ancestors likely did acquire the cytochrome oxidase gene.

### 3.2. Cytochrome Oxidase

In the Euryarchaeota, phylogenetic analysis of cytochrome oxidase subunit I (and the cytochrome oxidase I portion of genes with fused I and III subunits; Figure 2(a)) shows four well-supported clades. One clade is comprised of CoxAC (where cytochrome oxidase subunits I and III are fused) from *Picrophilus *and *Ferroplasma* (labeled as node 1 in the figure). Sequence characteristics and phylogenetic relationships show that these proteins belong to the Heme-copper oxygen reductase family A1 [23]. This phylogenetic pattern, in addition to ancestral state reconstruction (Figure 1), suggests that CoxAC was likely gained by an LGT event in the ancestor to *Picrophilus* and *Ferroplasma*. Three additional clades of cytochrome oxidase were observed in the Halobacteriales: CoxA1 (subunit I, family A1, node 2), CoxA2 (concatenated subunits I and III, family B, node 3), and CoxA3 (subunit I, family B, node 4). Most branching relationships within and between the clades showed moderate to poor support (Figure 2(a)). Ancestral state reconstruction suggests the presence of a cytochrome oxidase in the ancestor of the Halobacteriales (Figure 1). Ancestral state reconstruction using each form of the cytochrome oxidase genes predicts CoxA1 and CoxA3 (but not CoxA2, not shown) in the Halobacteriales ancestor. *Halobacterium *spp. formed the basal branch in the Halobacteriales in the genome tree. However, in the cytochrome oxidase tree, *Halobacterium *branched higher up in the CoxA1 and CoxA3 clades, suggesting that both sets of genes were acquired by independent lateral transfer events from donors outside the Euryarchaeota. A global phylogenetic tree containing families A and B sequences from Bacteria and Archaea (not shown; [23, 24]) shows CoxA1 and CoxA2 falling in a well-supported clade within family A1, and CoxA3 branching within family B. CoxA1 and CoxA2 did not branch as sister taxa with internal relationships consistent with the genome tree, which would be expected if the two were related by ancient gene duplication. CoxA2 homologs had a limited taxonomic distribution—only being observed in *Halorubrum, Haloterrigena, Haloarcula, Haloferax, *and *Halogeometricum.* Thus, CoxA2 was likely gained by an LGT event in the ancestor of these species (after the *Halobacterium* lineage diverged from the main Halobacteriales line of descent). Finally, one sequence from *Natronomonas pharaonis *(node 5) was rather divergent, branching with *Magnetospirillum *outside of any of the other archaeal clades. This is consistent with a recent lateral gene transfer event. In sum, five LGT events in the Euryarchaeota can be proposed to explain the phylogenetic pattern for cytochrome oxidase I and III (within the set of euryarchaeal taxa included in this study; Table 1).

Phylogenetic analysis of cytochrome oxidase subunit II in the Euryarchaeota (Figure 2(b)) similarly resulted in four clades. One clade contained *Picrophilus* and *Ferroplasma *(node 2). Examination of the physical location of the CoxB with CoxAC genes in the *Picrophilus *genome showed that they were linked, separated in the genome by a single open reading frame. The same observation was found for *Ferroplasma*. Thus, the LGT event that led to the acquisition of CoxAC also led to the acquisition of CoxB in the ancestor to *Picrophilus *and *Ferroplasma.* Three additional clades (CoxB1, CoxB2, and CoxB3; nodes 2–4) were found containing Halobacteriales taxa. Most branching relationships within and between the clades also showed moderate to poor support. Homologs to CoxB1 and CoxB3 (but not CoxB2) were observed in the two *Halobacterium* species. Again, *Halobacterium* CoxB1 and CoxB3 are found high up in the clade. Most genes in the CoxA1 and CoxB1 clades were physically linked, as were the genes for CoxA2 and CoxB2 and CoxA3 and CoxB3. Thus, the three LGT events that led to the acquisition of CoxA1, CoxA2, and CoxA3 also likely led to the acquisition of CoxB1, CoxB2, and CoxB3. The CoxB3 in *Natronomonas *(node 5) was also physically linked in the genome to CoxA3, that suggesting these two genes were acquired in a single LGT event as well. In sum, the total number of inferred LGT events for cytochrome oxidase subunits in the Euryarchaeota taxa included in this study is five (Table 1).

**Figure 3:**
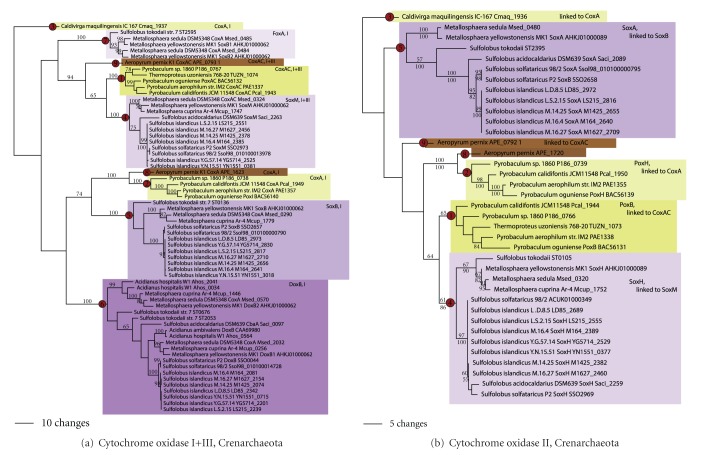
Cytochrome oxidase in the Crenarchaeota. Phylogenetic trees for (a) cytochrome oxidase subunit I and cytochrome oxidase subunits I+III and (b) cytochrome oxidase subunit II. Putative gene duplication events (e.g., leading to PoxB and PoxH) are indicated using black arrows.

Phylogenetic analysis of cytochrome oxidase in the Crenarchaeota (Figure 3(a)) was somewhat more complex. In the Thermoproteales, a fused CoxAC was found in *Pyrobaculum calidifontis, P. oguniense*, *P. *sp. 1860, *P. aerophilum, *and* Thermoproteus uzoniensis *(node 1). This protein falls under the Heme-copper oxygen reductase family A1 [23]. Another copy, CoxA, was found in *Pyrobaculum calidifontis, P. oguniense, P. *sp. 1860, and *P. aerophilum* (node 2). This second copy, a member of family B, branched separately from the CoxAC clade. Another CoxA in the family B was found in *Caldivirga* (node 3); however, this sequence did not branch with CoxA sequences from closely related *Pyrobaculum *spp. A global phylogenetic tree containing families A and B sequences from Bacteria and Archaea (not shown; [24]) showed *Pyrobaculum *CoxAC sequences branched with Sulfolobales SoxM sequences and family A1 sequences from Bacteria. *Pyrobaculum *CoxA sequences branched in a distinct location in the tree, in a well-supported clade with *Aeropyrum, *Halobacteriales CoxA3, and a wide diversity of bacterial family B sequences. The *Caldivirga *sequence branched with a phylogenetically distinct group of bacterial family B sequences from the Firmicutes and Proteobacteria. Relationships within the *Pyrobaculum *species of both clades, nevertheless, are congruent with the genome phylogeny (Figure 1). Thus, the simplest explanation for the observed phylogenetic pattern is three independent LGT events in the Thermoproteales: CoxA in *Caldivirga*, CoxA in the ancestor to* Pyrobaculum* spp., and CoxAC in the ancestor to *Pyrobaculum-Thermoproteus*. Three LGT events in the Thermoproteales were also inferred for *Caldivirga *and *Pyrobaculum* spp. using ancestral state reconstruction (Figure 1).

Four clades of cytochrome oxidases were observed in the Sulfolobales, corresponding to SoxM (fused subunits I and III, family A1, node 4), SoxB (subunit I, family B, node 5), DoxB (subunit I, family B, node 6), and a distinct clade in *Sulfolobus tokodaii *and *Metallosphaera *spp. (FoxA, family B, node 7). SoxM branched sister to *Pyrobaculum-Thermoproteus *CoxAC (65% and 100% support using MP and MB, resp.). This sister group relationship was also strongly supported in the global phylogeny containing Archaea and Bacteria (not shown), consistent with one LGT gain in the ancestor to the Sulfolobales. SoxB and DoxB formed a poorly to moderately well-supported clade with *Pyrobaculum* and *Aeropyrum *CoxA. However, branching relationships between and within the clades were often poorly or moderately supported. It is likely that SoxB and DoxB arose by a single LGT event followed by an ancestral gene duplication event (with subsequent duplications in *Metallosphaera *and *S. tokodaii*), given that branching relationships and the rooting of these two clades are consistent with the genome phylogeny. Nevertheless, phylogenetic trees with crenarchaeal cytochrome oxidases, crenarchaeal family B cytochrome oxidases, and the global family B tree are unresolved, and therefore, it cannot be formally ruled out that SoxB and DoxB were obtained by two independent LGT events. The FoxA clade is a newly identified clade of Heme-copper oxygen reductases found in *Sulfolobus tokodaii* and *Metallosphaera *spp. Transcriptional studies show that these genes are expressed under Fe(II)-oxidizing conditions [25]. Phylogenetic analysis shows that this sequence belongs to the family B of Heme-copper oxygen reductases; however, branching relationships between Sulfolobales SoxB, DoxB, and other archaeal cytochrome oxidases are unresolved (Figure 3(a), not shown). Thus, it is possible that FoxB arose by ancient gene duplication from a SoxB or DoxB ancestor, or it could have been gained independently via LGT. Thus, the minimal total number of LGT events involving cytochrome oxidases in the Sulfolobales is 2, while the maximum number of events is 4.

In the Desulfurococcales, CoxA and CoxAC (members of Heme copper oxygen reductase families B and A1, resp., nodes 8 and 9) were found in *Aeropyrum pernix*. Both branched with their respective homologs in distantly related *Pyrobaculum, *suggestive of two independent LGT events leading to two copies of cytochrome oxidase in *Aeropyrum. *This scenario is also predicted using ancestral state reconstruction (Figure 1). In summary, a minimum of  7 LGT events, and a maximum of 9, can be proposed to explain the phylogenetic pattern for cytochrome oxidase I and III in the Crenarchaeota (Table 1).

The phylogenetic tree for crenarchaeal cytochrome oxidase subunit II was similar to that for subunit I. Two clades containing *Pyrobaculum *spp. (PoxH and PoxB) were comprised of the same species, with similar branching relationships, to CoxAC and CoxA (nodes 1 and 2). The PoxH and PoxB genes were also physically linked to CoxA and CoxAC genes in the genome; thus, the two LGT events that likely led to the acquisition of CoxA and CoxAC in the ancestor to *Pyrobaculum* also led to the acquisition of PoxH and PoxB. The *Caldivirga* homolog branched separately from the *Pyrobaculum *clades, and this gene was physically linked to CoxA (evidence that the LGT event leading to the acquisition of *Caldivirga *CoxA also led to the acquisition of CoxB, node 3). In the Sulfolobales, two cytochrome oxidase subunit II clades were found (SoxA and SoxH, nodes 4 and 5). Again, branching relationships within the clades were only moderately or poorly supported; however, most of the genes for SoxA are physically linked to SoxB; thus, the LGT event leading to the acquisition of SoxB in the Sulfolobales also likely led to the acquisition of SoxA. Similarly, homologs in the SoxH clade are physically linked to SoxM; thus, the LGT leading to SoxM acquisition also likely transferred SoxH. Two *Aeropyrum* lineages are also seen in the cytochrome oxidase II tree, with branching relationships seen in the subunit I+III tree (nodes 8 and 9). One copy branched sister to the PoxH from *Pyrobaculum* and thus was likely acquired in the same LGT event that led to the acquisition of CoxA in *Aeropyrum. *The branching position of the second homolog was poorly supported. Nevertheless, this homolog is physically linked to the CoxAC gene in *Pyrobaculum*, and so it was most likely acquired in the same LGT event that led to the acquisition of CoxAC. In summary, seven LGT events can be proposed to explain the phylogenetic pattern for cytochrome oxidase II in the Crenarchaeota; however, these genes were obtained in the same LGT events that lead to the acquisition of cytochrome oxidase I and III.

### 3.3. Cytochrome bd-Type Quinol Oxidase

**Figure 4:**
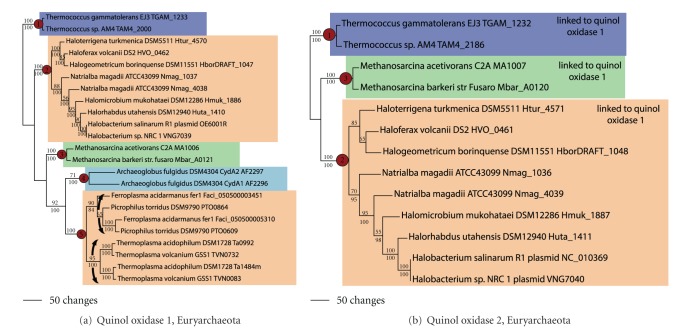
Cytochrome oxidase bd quinol oxidase in the Euryarchaeota. Phylogenetic trees for (a) quinol oxidase subunit 1 and (b) quinol oxidase subunit 2. Bootstrap values above 50% from MP analyses are shown above each branch; posterior probabilities from MB analyses are shown below each branch.

In the Euryarchaeota, cytochrome bd quinol oxidase subunit 1 fell into five clades (Figure 4(a)). The first clade (node 1) contained *Thermococcus gammatolerans *and *T. *sp. AM4, consistent with an LGT event in the ancestor to these closely related species. The second clade contained Halobacteriales taxa (node 2). *Halobacterium *in this clade branched high up in the clade, suggesting either that the LGT donor came from outside the Euryarchaeota or that euryarchaeal taxa higher up in the tree then became the donor for other euryarchaeal groups. The third clade (node 3) contained *Methanosarcina acetivorans *and *M. barkeri*, consistent with another LGT gain in the ancestor to these closely related species. The fourth clade (node 4) was comprised of two adjacent quinol oxidase copies in the *Archaeoglobus fulgidus *genome, suggestive of a single LGT gain followed by a gene duplication event. The fifth clade was comprised of Thermoplasmatales taxa (node 5). The phylogenetic pattern of duplicate copies in this clade is consistent with a single LGT gain followed by a gene duplication event in the ancestor to *Thermoplasma *spp. and a second duplication in the ancestor to *Ferroplasma *and *Picrophilus. *Ancestral state reconstruction (not shown) was consistent with five gains in the Euryarchaeota.

In the Euryarchaeota, cytochrome bd quinol oxidase subunit 2 fell into three clades (Figure 4(b)). The first clade (node 1) was comprised of *Thermococcus gammatolerans* and *T. *sp. AM4, the second (node 2) contained Halobacteriales taxa with branching relationships that were identical to those seen in the quinol oxidase 1 tree, and the third clade (node 3) contained *Methanosarcina acetivorans *and *M. barkeri*. Physical linkage between quinol oxidase 1 and 2 was observed for all euryarchaeal taxa; thus, the LGT events leading to the acquisition of quinol oxidase 1 in the Euryarchaeota also led to the simultaneous acquisition of quinol oxidase 2.

**Figure 5:**
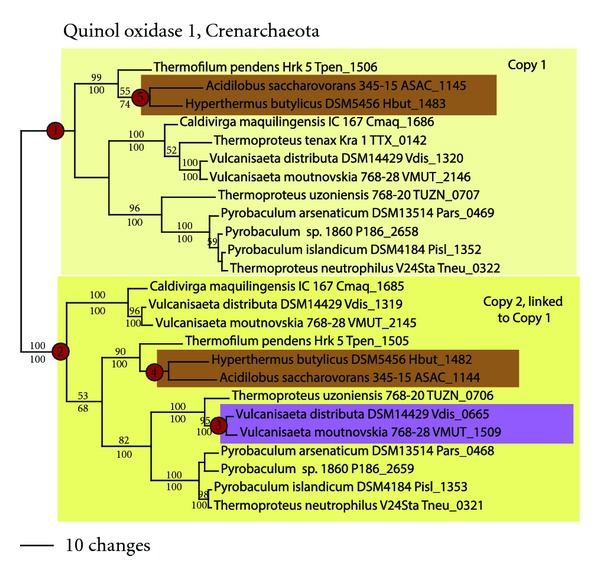
Cytochrome bd quinol oxidase in the Crenarchaeota. Phylogenetic tree for quinol oxidase subunit 1.

In the Crenarchaeota, two copies of cytochrome bd quinol oxidase 1, forming sister clades, were found in *Hyperthermus, Acidilobus,* and most Thermoproteales taxa (Figure 5, nodes 1 and 2). Additional copies also were found in *Vulcanisaeta distributa *and *V. moutnovskia *(node 3). Phylogenetic analyses of the Thermoproteales taxa showed that neither sister clade was rooted with *Thermofilum* (as seen in the genome phylogeny); however, analyses of each sister clade in isolation (not shown) resulted in a tree that was congruent with the genome phylogeny. While this appears to be consistent with two independent LGT events in the ancestor to the Thermoproteales, examination of the genome positioning shows that both copies of quinol oxidase 1 in the Thermoproteales are adjacent in the genome. Thus, the most parsimonious explanation is a single LGT event that transferred two tandem, but distantly related, copies of quinol oxidase into the ancestor of the Thermoproteales. Two tandem copies of quinol oxidase 1 were also seen in *Hyperthermus* and *Acidilobus* (nodes 4 and 5), both branching sister to *Thermofilum. *The most likely explanation is a single LGT event, possibly from *Thermofilum,* which transferred the two tandem copies into *Hyperthermus-Acidilobus* (it is unlikely that the ancestor to *Hyperthermus-Acidilobus *was the donor for the Thermoproteales quinol oxidases, since the Thermoproteales clade is significantly older; Figure 1). One additional LGT event likely led to the acquisition of the third quinol oxidase copy in *Vulcanisaeta *spp. (node 3). Ancestral state reconstruction (not shown) was consistent with three independent LGT events in the Crenarchaeota.

### 3.4. Dissimilatory Sulfite Reduction

**Figure 6:**
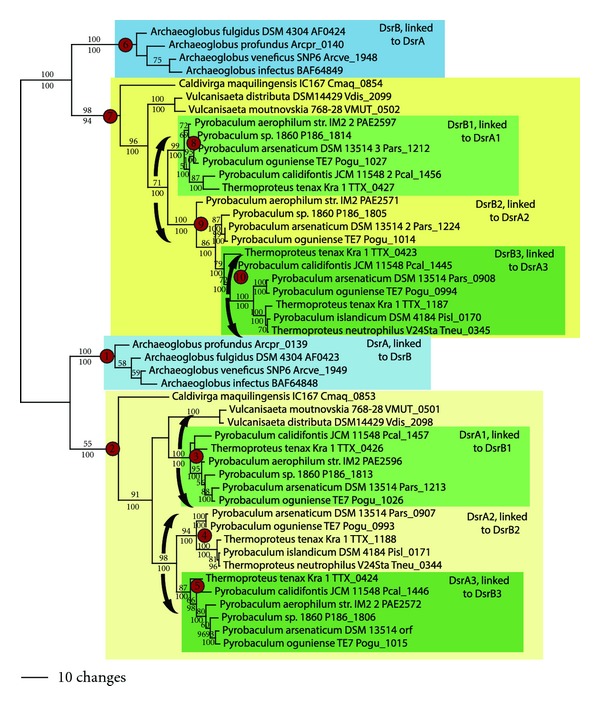
Dissimilatory sulfite reduction. Phylogenetic tree for DsrA and DsrB.

DsrA and DsrB are related proteins that derived from an ancient gene duplication event [26]. The phylogeny of DsrA and DsrB (Figure 6) shows two well-supported clades in the archaeal domain, one containing *Archaeoglobus *spp. (nodes 1 and 6) and the second containing *Caldivirga, Vulcanisaeta, Pyrobaculum, *and *Thermoproteus *species (nodes 2 and 7). Genomic positioning shows that DsrA is linked to DsrB in all species, and one copy of DsrA and DsrB is found in *Caldivirga* and *Vulcanisaeta* spp. However, three copies are found in most of the genomes of *Pyrobaculum* and *Thermoproteus* spp., all forming a monophyletic group (nodes 3–5 and 8–10). This suggests that multiple gene duplication events occurred involving DsrAB in the ancestor to the *Pyrobaculum-Thermoproteus* clade, followed by later gene losses in some taxa. Thus, two LGT events are postulated for DsrAB in the archaea: once in the Archaeoglobales and once early in history of the Thermoproteales (Table 2). This is consistent with ancestral state reconstruction of the presence of DsrAB (not shown).

### 3.5. Thiosulfate Oxidation

**Figure 7:**
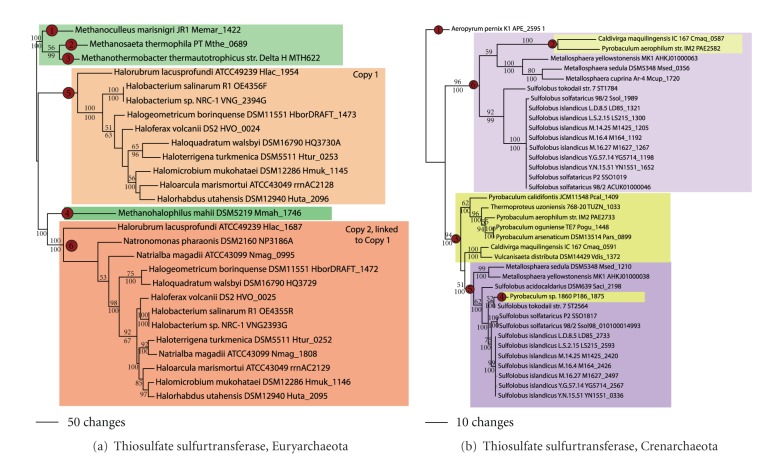
Thiosulfate reduction. Thiosulfate sulfurtransferase (SseA) phylogeny for (a) the Euryarchaeota and (b) Crenarchaeota.

Thiosulfate sulfurtransferase (SseA) catalyzes the oxidation of thiosulfate to sulfite, while reducing cyanide to thiocyanate [27]. The phylogeny of the alpha subunit in the Euryarchaeota (Figure 7(a)) showed two clades of methanogen sequences (nodes 1–4) and two clades of sequences from the Halobacteriales (nodes 5 and 6). Little is known about thiosulfate metabolism in halophilic archaea; however, growing evidence suggests that many strains are able to oxidize it to sulfite and subsequently reduced to sulfide [28, 29]. The four methanogen sequences were found in distantly related euryarchaeal lineages. This provides evidence for four independent LGT events in the methanogens and is consistent to the pattern observed by ancestral state reconstruction (not shown). The Halobacteriales thiosulfate sulfurtransferase fell into two clades (copies 1 and 2), neither of which was rooted with *Halobacterium. *The sequences from copy 1 were found to be immediately adjacent to sequences from copy 2, suggesting that the two copies have been inherited as a single unit. Ancestral state reconstruction shows a high likelihood that SseA was present in the ancestor of the Halobacteriales (not shown).

In the Crenarchaeota (Figure 7(b)), thiosulfate sulfurtransferase was found in one member of the Desulfurococcales (*Aeropyrum*), many Thermoproteales, and many of the Sulfolobales. Thiosulfate stimulates growth in *Aeropyrum* and *Pyrobaculum oguniense* [30, 31], can be oxidized by *Pyrobaculum aerophilum *[32], and serves as an electron acceptor in *Caldivirga, Thermoproteus uzoniensis, *and many *Pyrobaculum *spp. [33, 34]. A single LGT event most likely led to the acquisition of SseA in *Aeropyrum *(node 1). The Thermoproteales sequences fell into three clades, one containing *Caldivirga *and *P. aerophilum *(node 2), another clade containing* Caldivirga, Vulcanisaeta distributa, Thermoproteus uzoniensis*, and *Pyrobaculum *spp. (node 3), and a clade containing *P. *sp. 1860 (node 4). Two LGT events may have led to the acquisition of two distantly related SseA copies in the ancestor to *Caldivirga* and *Pyrobaculum*, with a third acquisition in *P*. sp. 1860. The Sulfolobales sequences fell into two clades (nodes 5 and 6), both of which were rooted with *Metallosphaera. *Thus, two LGT events likely led to the acquisition of two distantly related SseA copies in the ancestor to the Sulfolobales. The inferred number of LGT events of thiosulfate oxidation in both the Euryarchaeota and Crenarchaeota is 11.

### 3.6. Sulfur Oxidation and Reduction

**Figure 8:**
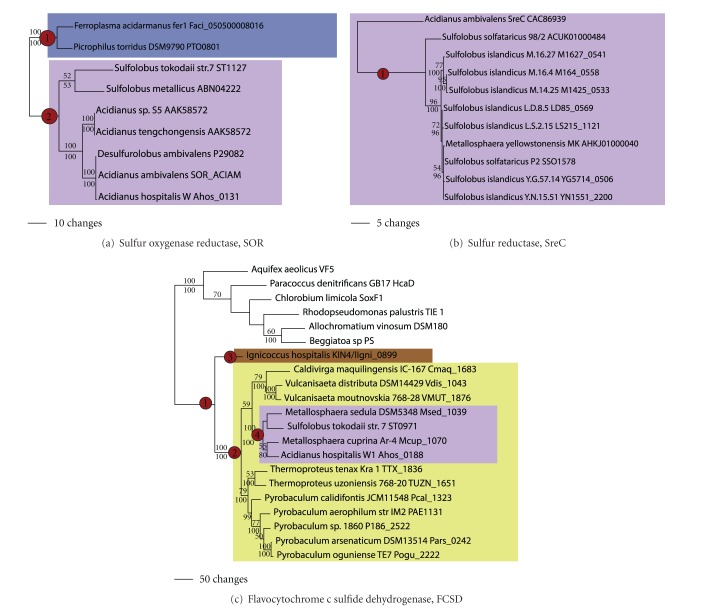
Sulfur oxidation and reduction. Phylogenetic trees for (a) SOR sulfur oxygenase reductase, (b) SreC sulfur reductase, and (c) FCSD flavocytochrome c sulfide dehydrogenase.

Sulfur oxygenase reductase (SOR) catalyzes the aerobic disproportionation of sulfur into sulfide and bisulfite. SOR activity in the archaea has been demonstrated for a number of Sulfolobales taxa [3] and more recently in *Acidianus tengchongensis *[35]. Homologs were also found in *Sulfolobus tokodaii, S. metallicus, Acidianus, *and* Desulfurolobus*. Phylogenetic analysis (Figure 8(a)) shows that these sequences all form a monophyletic group, and thus, SOR was likely gained in the Sulfolobales (node 1). In the Euryarchaeota, putative homologs to SOR were found in the genomes of *Ferroplasma *and *Picrophilus* (node 2). Given that these two species are closely related, it is likely that SOR was also acquired via LGT in the ancestor to these two taxa.

Sulfur reductase catalyzes the reduction of sulfur or polysulfide to hydrogen sulfide. In the archaeal domain, the SreABCDE gene cluster has been demonstrated in *Acidianus* to carry out the reduction of sulfur [36]. Putative homologs have been found in the members of *Sulfolobus* species (node 1). Their phylogenetic relationships (Figure 8(b)) were congruent with the genome phylogeny; thus, sulfur reductase was likely acquired in the ancestor to the Sulfolobales.

Flavocytochrome c sulfide dehydrogenase (FCSD) in Bacteria results in the anaerobic oxidation of sulfide to sulfur. Homologs to this FCSD have been identified in a number of Archaea (including *Ignicoccus, Caldivirga, Pyrobaculum, Vulcanisaeta, Thermoproteus, Metallosphaera, Acidianus, *and *Sulfolobus tokodaii*); however, FCSD activity has yet to be demonstrated in the archaeal domain. Phylogenetic analysis shows that archaeal FCSD forms a monophyletic group (node 1, Figure 8(c)) with a sister group relationship with bacterial proteins with demonstrated FCSD activity. Sequences from the Thermoproteales taxa *Caldivirga, Pyrobaculum, Thermoproteus, *and *Vulcanisaeta *formed a monophyletic group (node 2), with branching relationships consistent with the genome phylogeny, and ancestral state reconstruction predicted the presence of this gene in the ancestor of these taxa. An FCSD homolog was also found in *Ignicoccus*—this was likely gained by an independent LGT event (node 3). Similarly, the FCSD homolog in *Acidianus*, *Metallosphaera*, and *Sulfolobus tokodaii* formed a monophyletic group (node 4), so their common ancestor also likely gained this gene by LGT. 

**Figure 9:**
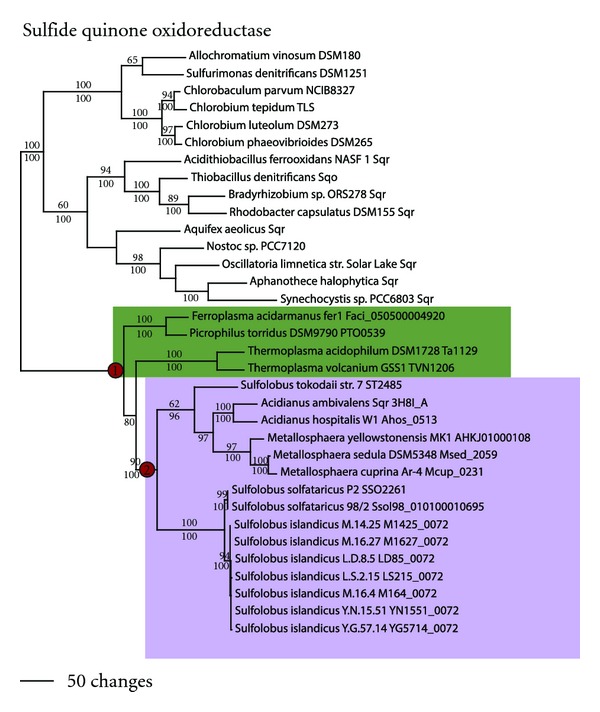
Anaerobic sulfide oxidation. Sulfide quinone oxidoreductase (SQO) phylogeny for (a) Euryarchaeota and (b) Crenarchaeota.

Sulfide quinone oxidoreductase (SQO) also catalyzes the anaerobic oxidation of sulfide to sulfur and shows sequence similarity to FCSD as well as to a large number of widely distributed putative homologs annotated as FAD-dependent pyridine-nucleotide disulfide oxidoreductases. In the Euryarchaeota, SQO is found in the Thermoplasmatales (node 1, Figure 9). Phylogenetic analyses and ancestral state reconstruction suggested that SQO was acquired by an LGT event in the ancestor of this group. In the Crenarchaeota, SQO (including the SQO from *Acidianus *with demonstrated activity) was found in the Sulfolobales, and the branching patterns in this group were congruent with the genome phylogeny (node 2). Thus, SQO in the Sulfolobales was likely gained in the ancestor to this group. Archaeal SQO homologs formed a monophyletic group sister to demonstrated SQO homologs in the bacterial domain, distinct from FCSD and the putative pyridine-dinucleotide disulfide oxidoreductases (not shown).

### 3.7. Nitrate and Nitrite Reduction

**Figure 10:**
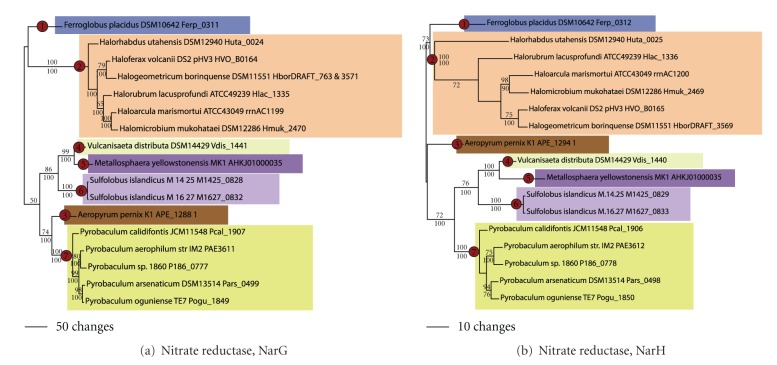
Nitrate reduction. Phylogenetic trees for nitrate reductase (a) catalytic alpha subunit (NarG) and (b) electron transfer beta subunit (NarH).

Nitrate reductase reduces nitrate to nitrite during anaerobic respiration. The catalytic and electron transfer subunits of nitrate reductase (Figures 10(a), and 10(b)) in the Euryarchaeota were found in *Ferroglobus *(node 1) and in a clade that contains several Halobacteriales taxa (node 2). In the Crenarchaeota nitrate, reductase subunits were found in the distantly related taxa *Aeropyrum* (node 3)*, Vulcanisaeta distributa *(node 4)*, Metallosphaera yellowstonensis* (node 5), two strains of *Sulfolobus islandicus *(node 6), and a clade containing *Pyrobaculum* spp. (node 7). Because nitrate reductase was not found in *Halobacterium *spp., it is likely that the latter acquisition occurred after diversification of *Halobacterium. *The phylogenetic patterning of the nitrate reductase catalytic subunit (Figure 10(a)) is consistent with seven LGT events. The phylogenies of NarG and NarH were identical, and the two genes were adjacent in the genomes of all taxa. Thus, the LGT events leading to the acquisition of NarG also likely led to the acquisition of NarH (Table 3).

**Figure 11:**
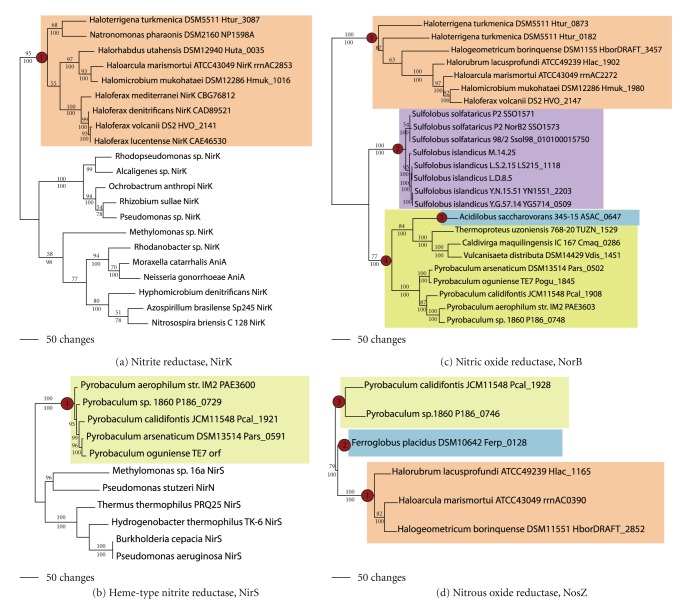
Nitrite reduction. Phylogenetic trees for (a) Cu-containing nitrite reductase beta subunit (NirK), (b) Heme-containing nitrite reductase (NirS), (c) nitric oxide reductase (NorB), and (d) nitrous oxide reductase (NosZ).

Dissimilatory nitrite reductases (NirK) reduce nitrite to either nitric oxide or nitrous oxide. Copper-type nitrite reductases have been identified in some Halobacteriales taxa (Figure 11(a); [37]). Phylogenetic analyses show that they fall into a monophyletic group (node 1). The phylogenetic pattern suggests that they could have been acquired once, likely after the diversification of *Halobacterium* spp. However, ancestral state reconstruction suggests that two independent gains could have also occurred (nodes 1 and 2), once being in the ancestor to *Haloferax* and *Halogeometricum.*

Homologs to bacterial Heme-type nitrite reductases (NirS) have also been identified in the genomes of three *Pyrobaculum* species. These also formed a well-supported monophyletic group (node 1, Figure 11(b)). Each of the *Pyrobaculum *sequences is linked to cytochrome c proteins (not shown) that form part of the nitrite reductase complexes in bacteria. Thus, a single LGT event likely led to the acquisition of Heme-type nitrite reductases in the ancestor to *Pyrobaculum *spp.

Nitric oxide reductase (NorB) reduces nitric oxide to nitrous oxide. Phylogenetic analysis showed four well-supported clades of NorB in the archaeal domain (Figure 11(c)). One clade contained the Halobacteriales (but not *Halobacterium*, node 1), another contained *S. solfataricus* and *S. islandicus *species (node 2), a third contained the genus *Acidilobus* (node 3), and the fourth (ode 4) contained several taxa in the Thermoproteales (*Caldivirga, Vulcanisaeta distributa, Thermoproteus uzoniensis, *and several *Pyrobaculum *spp.).This is consistent with four lateral transfer events.

Finally, nitrous oxide reductase (NosZ) reduces nitrous oxide to nitrogen. NosZ homologues (Figure 11(d)) were found in only a few archaeal taxa: *Haloarcula, Halobacterium, Halobiforma, Haloferax, Halogeometricum, Halopiger, *and *Halorubrum* in the Halobacteriales (node 1), *Ferroglobus* (node 2), and two species of *Pyrobaculum* (node 3). The phylogenetic pattern was consistent with three lateral transfer events: once in the Halobacteriales, once in *Ferroglobus*, and once in the clade containing *Pyrobaculum *spp.

### 3.8. Nitrogen Fixation

**Figure 12:**
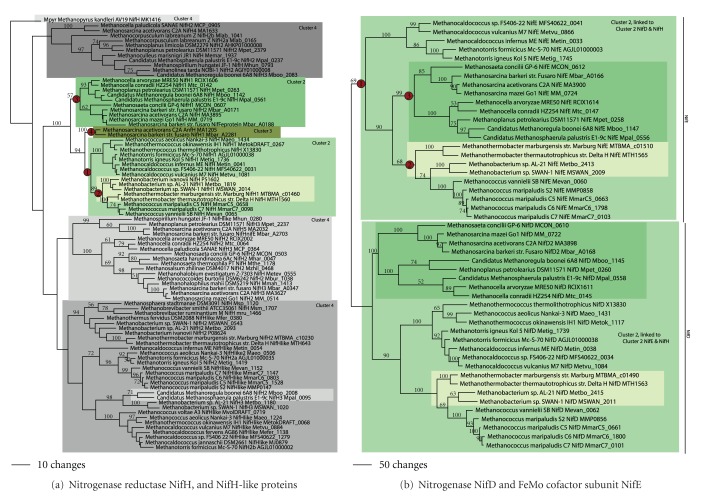
Nitrogen fixation. Phylogenetic trees for (a) nitrogenase reductase NifH and NifH-like proteins (type 4) and (b) the nitrogenase alpha subunit NifD with the FeMo cofactor subunit NifE.

As has been noted by many investigators, the distribution of nifH-like genes (making up “cluster 4”; Figure 12(a)) is widespread among methanogens, including the basal lineage *Methanopyrus*. These proteins have recently been shown to form a complex with NifD-like proteins, yet they have no apparent role in nitrogen fixation [38]. True NifH genes involved in nitrogen fixation, however, show a much smaller taxonomic range. Indeed, the ability to fix nitrogen is found only in a small number of methanogens (*Methanococcus maripaludis, M. thermolithotrophicus, M. aeolicus, M. vannielii, Methanothermobacter thermoautotrophicum, Methanosarcina barkeri, Methanospirillum, *and *Methanocaldococcus *FS406-22 [32, 39, 40]). Many investigators have inferred a significant role for LGT as well as gene duplication events in the evolutionary history of archaeal nitrogen fixation genes, while a cursory look at the NifH phylogenetic tree (Figure 12(a)) is consistent with previous proposals that nitrogenase first arose in the methanogens [41]. A more detailed analysis suggests that this may not be the case.

Nitrogenase reductases (NifH) fell into a well-supported clade comprising “clusters 2 and 3” (Figure 12(a), clades outlined in green) to the exclusion of proteins related to NifH that constitute “cluster 4” (clades outlined in gray). Within “cluster 2” sequences, an AU test showed that the genome phylogeny and the NifH phylogeny for the Methanococcales were not significantly different (*P* = 0.068). Relationships within and between the Methanomicrobiales and *Methanosarcina *were identical to the genome phylogeny. In contrast, an AU test showed that the “cluster 2” phylogeny (including Methanococcales, Methanobacteriales, Methanomicrobiales, and Methanosarcinales) was significantly different from the genome phylogeny (*P* = 2*e* ^−5^). The point of major incongruence was in the positioning of Methanobacteriales with respect to the Methanococcales and the rooting of the Methanococcales. In addition, “cluster 3” sequences showed positioning that was different from the genome tree. This suggests four lateral transfer events of nitrogenase: once in the ancestor to the Methanococcales (node 1), once in the Methanobacteriales (node 2), once in the ancestor to the Methanomicrobiales and Methanosarcinales (node 3), and once again in *Methanosarcina* spp. (resulting in the acquisition of the alternative nitrogenases of “cluster 3”; node 4).

The nitrogenase alpha subunit (NifD) and the FeMo cofactor subunit (NifE) are related to one another, likely as a result of an ancestral gene duplication event [42]. Observations of relationships in these trees (Figure 12(b)) as well as AU tests (not shown) revealed relationships that were not significantly different from the relationships observed in the NifH tree. In terms of genomic positioning, “cluster 2” NifH is linked to “cluster 2” NifD and NifE. This suggests that when NifH was acquired by LGT, so were NifD and NifE (Table 3).

### 3.9. Degradation of Select Organocompounds

**Figure 13:**
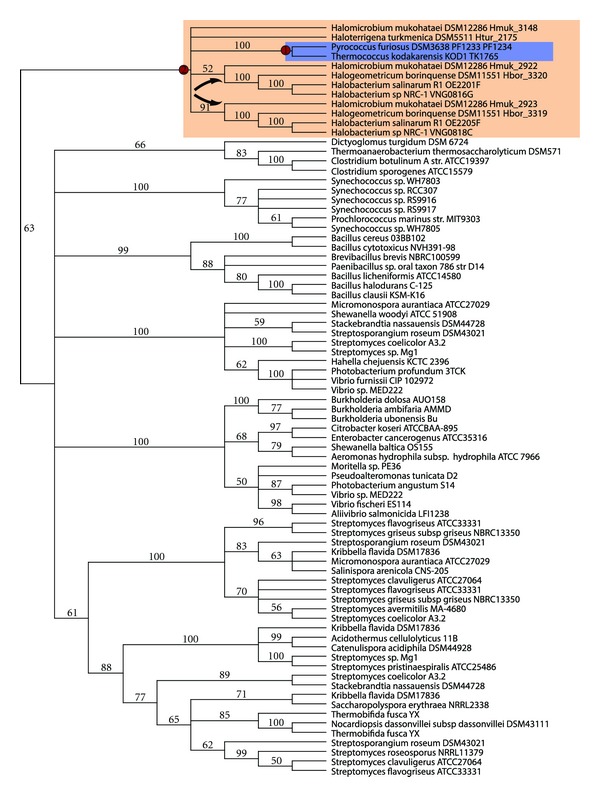
Chitinase. Phylogenetic tree for glycosyl hydrolase family 18 ChiA.

Most Thermococcales cultures have never been tested for chitin or chitosan (a form of deacetylated chitin) degradation [43, 44]; however, these organisms do inhabit deep sea hydrothermal vent ecosystems which harbor abundant chitin-containing metazoans (such as giant tube worms, crabs, and shrimp). Two genes with sequence similarity to known chitinases are present in the genomes of *Pyrococcus furiosus* and *Thermococcus kodakarensis. *Biochemical studies demonstrated that these genes exhibit chitinase activity [45, 46]. The chitinase genes are part of a unique chitin degradation pathway that includes at least three additional unique genes (exo-*β*-D-glucosaminidase, glucosamine-6-phosphate deaminase, and diacetylchitobiose deacetylase) [47, 48]. Phylogenetic analyses demonstrated that chitinases in the Thermococcales formed a well-supported monophyletic group (node 1, Figure 13), as did the three additional genes involved in chitin degradation (now shown). The chitinase and chitin degradation genes are all closely spaced along the genome. Thus, there was likely a single LGT event that led to the acquisition of chitin degradation genes in the ancestor to the Thermococcales (Table 4).

Many Halobacteriales also have chitinase-like genes. The halophilic archaea are not generally considered to be chitin degraders, however a recent study has demonstrated that Halobacterium sp. NRC-1 is capable of degrading chitin [49] and they live in environments where brine shrimp are often abundant. Thus, it is possible that, as in the Thermococcales, chitin degradation has been underestimated in the Halobacteriales. Several copies of chitinases are seen in the genomes of many Halobacteriales taxa (node 2, Figure 13), nevertheless they all appear to form a monophyletic group, and the multiple copies are often adjacent or nearby in the genome. Two subclades were seen to have mirror relationships, suggesting that two copies of chitinases were obtained by LGT in the ancestor of the Halobacteriales. Neither of these subclades, however, is rooted with *Halobacterium *(as seen in the genome phylogeny), and thus the two subclades likely did not arise as a result of ancestral or recent gene duplication. Rather the two copies were likely acquired by the same LGT event from a donor that came outside of the Thermococcales or Halobacteriales. This was then likely followed by losses of chitinase genes in other taxa in the Halobacteriales, including *Natrialba, Halorubrum, Haloquadratum, Natronomonas, Halorhabdus, *and *Haloarcula.*

**Figure 14:**
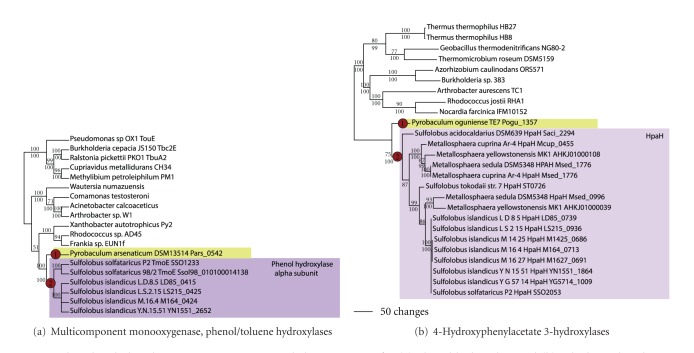
Phenol and phenylacetate monooxygenases. Phylogenetic trees for (a) phenol hydroxylase and (b) 4-hydroxyphenylacetate 3-hydroxylase.

**Figure 15:**
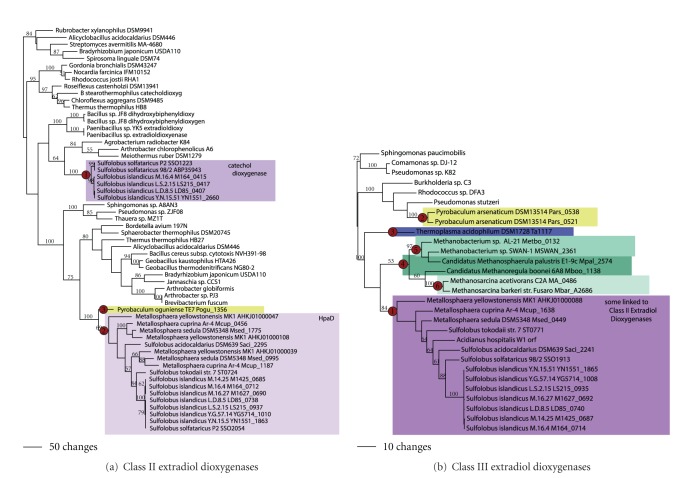
Extradiol dioxygenases. Phylogenetic trees for (a) class II extradiol dioxygenases and (b) class III extradiol dioxygenases.

Protein sequences related to those that catalyze the degradation of phenolic compounds, such as phenol, catechol, and phenylacetate, were found in the genomes of several archaeal groups (Figures 14 and 15). In the bacterial domain, members of class II extradiol dioxygenases have been shown to be capable of adding hydroxyl groups to the phenolic rings of catechol and hydroxyphenylacetate. *Sulfolobus solfataricus* P2 has been demonstrated to be able to use phenol, catechol, and toluene as sole carbon sources [50]; however, its close relative *S. solfataricus* 98/2 cannot be due to an insertion element in the operon containing phenol hydroxylase (which is also transcribed only in the presence of phenol; [51]). Archaeal relatives of multicomponent monooxygenases that hydroxylate phenol, toluene, and xylene have been observed in *Pyrobaculum arsenaticum, Sulfolobus solfataricus* strains, and several strains of *Sulfolobus islandicus *(Figure 14(a)). The phylogenetic pattern suggests a single LGT acquisition in *Pyrobaculum arsenaticum* (node 1), and a second acquisition in the ancestor to *S. solfataricus-S. islandicus* (node 2).

Relatives of monooxygenases that are involved in the hydroxylation of 4-hydroxyphenylacetate were also observed in the Sulfolobales, annotated as HpaH (Figure 14(b)). These sequences formed a well-supported clade and were found in *Metallosphaera *spp*., S. tokodaii, S. acidocaldarius, *one strain of *S. solfataricus *(P2), and all strains of *S. islandicus. *This suggests two LGT acquisition events: once in *Pyrobaculum oguniense* (node 1) and once again in the ancestor to the Sulfolobales (node 2).

Relatives of class II extradiol dioxygenases that are involved in cleavage of the phenolic rings of catechol and dihydroxyphenol acetate (Figure 15(a)) have been found in the genomes of the Sulfolobales. These sequences were observed to fall into two distinct well-supported clades. One clade (node 1) comprised both strains of *Sulfolobus solfataricus* and four strains of *S. islandicus*. Biochemical analyses have shown the proteins in both strains of *S. solfataricus* function as a catechol 2,3 dioxygenase and 4-chlorocatechol dioxygenase [50, 51]. The taxonomic distribution in this clade was identical to that found in the clade of phenol hydroxylases in the Sulfolobales (node 2, Figure 14(a)), their phylogenetic trees were identical, and, indeed, the proteins were found to be located nearby in the genome in all taxa. The second clade (node 2), annotated as HpaD, was more widespread in the Sulfolobales being present in *Metallosphaera *spp. (two to three copies), *S. tokodaii, S. acidocaldarius, S. solfataricus* P2,and all strains of *S. islandicus*. A third ortholog was identified in *Pyrobaculum oguniense *(node 3). Although the function of the archaeal HpaD ortholog has yet to be demonstrated, phylogenetic analyses suggest its functions as a 3,4-dihydroxyphenyl acetate dioxygenase [50]. All HpaH sequences in the Sulfolobales genomes were adjacent to the HpaD sequences in class II extradiol dioxygenases (Figure 14(a)). Relationships within the catechol dioxygenase and HpaD clades showed only a moderate or poor support; branching relationships however were similar to those observed in the genome phylogeny.

Relatives of class III extradiol dioxygenases were also found in the genomes of the Sulfolobales, *Thermoplasma acidophilum, Pyrobaculum arsenaticum*, and three small groups of distantly related methanogens (Figure 15(b)). The biochemical function of these proteins in the archaeal domain is presently not known. A well-supported clade (node 1) contained relatives from *Metallosphaera, S. tokodaii, S. acidocaldarius, S. solfataricus *98/2, and all strains of *S. islandicus.* Again, relationships within the clade were only moderately or poorly supported; however, branching relationships were similar to those in the genome phylogeny suggesting a single lateral transfer event in the ancestor to the Sulfolobales. Interestingly, the genes from all *S. islandicus* strains were found to be nearby the class II HpaD extradiol sequences, but such juxtaposition was not observed for any other taxa in the Sulfolobales. A homolog is also found in *Thermoplasma acidophilum* (node 3), consistent with a single gain in this taxon. A lone homolog to class III extradiol dioxygenase was also found in *Pyrobaculum arsenaticum* (node 3, Figure 15(b)), located in a similar location in the genome as the protein for phenol hydroxylase (node 1; Figure 14(b)), and both branch sisters suggesting they arose by a common LGT event followed by gene duplication. The phylogenetic distribution of the methanogen sequences suggests independent LGT events into three distantly related groups of methanogens (nodes 4–6). In sum, the phylogenetic pattern suggests six independent gains of class III extradiol dioxygenases by LGT events.

### 3.10. Rates of Lateral Transfer

Table 5 summarizes the number of LGT events per gene and converts this to a rate of number of LGT events over the age span of the archaeal domain (approximately 3.5 billion years). The gene with the highest transfer rate was thiosulfate sulfurtransferase (SseA), calculated to have been transferred 3.1 times per billion years across the entire archaeal domain of life. Cytochrome oxidase, quinol oxidase, and the genes coding for the oxidation of phenolic compounds were also transferred at higher rates (2.6–3.4 events per billion years). Most of other genes showed a small number of lateral transfer events, amounting to less than one transfer event per billion years. This suggests that gene acquisitions, and hence changes in the metabolic niches of archaea, have been slowly changing over geologic time. This observation is consistent with the complexity hypothesis [4–7].

## 4. Conclusions

Although metabolic genes are often considered to have been frequently swapped between prokaryotic lineages via the process of lateral gene transfer, a phylogenomic approach using explicit phylogenetic reconstruction and ancestral state reconstruction suggests that lateral transfer events involving metabolic genes are rare, across deep geologic time. These stable transfers likely occurred only a small number of times on the order of <1–3 transfer events per billions of years. 

## Figures and Tables

**Table 1:** Aerobic respiration.

Gene name	Gene	Linked to	Number of LGT events	Monophyletic clades
Cytochrome c oxidase subunit I and III	CoxA CoxAC SoxB DoxB SoxM	CoxB in *Ferroplasma *and *Picrophilus* linked to CoxAC CoxB1 in the Halobacteriales linked to CoxA1 CoxB2 in the Halobacteriales linked to CoxA2 CoxB3 in the Halobacteriales linked to CoxA3 CoxB in *Natronomonas* PoxH in Thermoproteales and *Aeropyrum *linked to CoxA PoxB in *Pyrobaculum* spp. And *Aeropyrum *linked to CoxAC SoxA in the Sulfolobales linked to SoxB SoxH in the Sulfolobales linked to SoxM	12–14	(1) *Ferroplasma, Picrophilus* (2) Halobacteriales (CoxA1) (3) Halobacteriales (CoxA2) (4) Halobacteriales (CoxA3) (5) *Natronomonas* (6) *Caldivirga* (7) *P.* spp. (CoxA) (8) *P.* spp., *Thermoproteus uzoniensis *(CoxAC) (9) *Aeropyrum* (CoxA) (10) *Aeropyrum* (CoxAC) (11) Sulfolobales (SoxB) [(12) Sulfolobales (DoxB)]^*∧*^ [(13) Sulfolobales (FoxA)]^*∧*^ (14) Sulfolobales (SoxM)

Cytochrome c oxidase subunit II	CoxB PoxB PoxH SoxA SoxH	CoxB in *Ferroplasma *and *Picrophilus* linked to CoxAC CoxB1 in the Halobacteriales linked to CoxA1 CoxB2 in the Halobacteriales linked to CoxA2 CoxB3 in the Halobacteriales linked to CoxA3 CoxA3 in *Natronomonas* PoxH in Thermoproteales and *Aeropyrum *linked to CoxA PoxB in *Pyrobaculum* spp. and *Aeropyrum *linked to CoxAC SoxA in the Sulfolobales linked to SoxB SoxH in the Sulfolobales linked to SoxM	(11)*	(1) *Ferroplasma, Picrophilus* (2) Halobacteriales (CoxB1) (3) Halobacteriales (CoxB2) (4) Halobacteriales (CoxB3) (5) *Caldivirga* (6) *P.* spp. (PoxH) (7) *P. aerophilum* spp., *Thermoproteus uzoniensis* (PoxB) (8) *Aeropyrum* (PoxH) (9) *Aeropyrum* (PoxB) (10) Sulfolobales (SoxA) (11) Sulfolobales (SoxH)

Quinol oxidase subunit I		Linked to quinol oxidase subunit II in *Methanosarcina, Thermococcus, *and Halobacteriales	8	(1) *T. gammatolerans, T. *sp. AM4 (2) Halobacteriales (3) *M. acetivorans, M. Barkeri* (4) *Archaeoglobus* (CydA1 and CydA2) (5) *Ferroplasma, Picrophilus, Tp. acidophilum, Tp. Volcanium* (6) Thermoproteales (copy 1 and copy 2) (7) *Hyperthermus* and *Acidilobus *(copy 1 and copy 2) (8) *Vulcanisaeta* spp. (extra copy)

Quinol oxidase subunit II		Linked to quinol oxidase subunit I in *Methanosarcina, Thermococcus, *and Halobacteriales	(3)	(1) *T. gammatolerans, T. *sp. AM4 (2) *M. acetivorans, M. Barkeri* (3) Halobacteriales

^∗^Numbers in brackets indicate that these genes are linked to the previously listed gene and therefore should not be counted as an independent LGT event.

^*∧*^Taxa in brackets indicate that these genes may have arisen by ancient gene duplication, and not LGT.

**Table 2:** S metabolism.

Gene name	Gene	Linked to	Number of LGT events	Monophyletic clades
Dissimilatory sulfite reductase, alpha subunit	DsrA	DsrB may be linked to SseA and SQR in *Pyrobaculum arsenaticum* and *P. calidifontis *	2	(1) *Archaeoglobus *spp. (2) *Caldivirga*, *Vulcanisaeta, Pyrobaculum* spp., and *Thermoproteus *

Dissimilatory sulfite reductase, beta subunit	DsrB	DsrA may be linked to SseA and SQR in *Pyrobaculum arsenaticum* and *P. calidifontis *	(2)	(1) *Archaeoglobus *spp. (2) *Caldivirga*, *Vulcanisaeta, Pyrobaculum* spp., and *Thermoproteus *

Thiosulfate sulfurtransferase	SseA	May be linked to DsrAB and SQR in *Pyrobaculum arsenaticum* and *P. calidifontis *	11	(1) *Methanohalophilus* (2) *Methanosaeta* (3) *Methanoculleus* (4) *Methanothermobacter* (5) Halobacteriales (6) *Aeropyrum* (7) *Caldivirga*,* P. Aerophilum* (8) *Caldivirga*,* Pyrobaculum* spp., *Thermoproteus uzoniensis,* and *Vulcanisaeta* (9) *Pyrobaculum* sp. 1680 (10) Sulfolobales (11) Sulfolobales

Sulfur oxygenase reductase	SOR		2	(1) *Ferroplasma, Picrophilus* (2) *Acidianus* spp., *Desulfurolobus, S. metallicus, S. tokodaii *

Sulfur reductase	SreC		1	(1) Sulfolobales

Flavocytochrome c sulfide dehydrogenase	FCSD		3	(1) *Ignicoccus* (2) *Caldivirga, Vulcanisaeta, Pyrobaculum* spp., and *Thermoproteus* spp. (3) *Metallosphaera, S. tokodaii,* and *Acidianus *

Sulfide quinone oxidoreductase	SQO		2	(1) *Ferroplasma, Picrophilus, Tp. acidophilum,* and *Tp. Volcanium* (2) Sulfolobales

**Table 3:** Nitrogen metabolism.

Gene name	Gene	Linked to	Number of LGT events	Monophyletic clades
Nitrate reductase, alpha subunit	NarG	NarH, NorB in *Pyrobaculum *spp.	7	(1) *Ferroglobus* (2) *Haloarcula, Haloferax, Halogeometricum, Halomicrobium, Halorhabdus,* and *Halorubrum* (3) *Aeropyrum* (4) *P. aerophilum, P. arsenaticum,* and *P. Calidifontis* (5) *S. islandicus* M.14.25, *S. islandicus* M.16.27 (6) *Metallosphaera yellowstonensis* (7) *Vulcanisaeta distributa *

Nitrate reductase, beta subunit	NarH	NarG, NorB in *Pyrobaculum *spp.	(7)	(1) *Ferroglobus* (2) *Haloarcula, Haloferax, Halogeometricum, Halomicrobium, Halorhabdus,* and *Halorubrum* (3) *Aeropyrum* (4) *P. aerophilum, P. arsenaticum,* and *P. Calidifontis* (5) *S. islandicus* M.14.25, *S. islandicus* M.16.27 (6) *Metallosphaera yellowstonensis* (7) *Vulcanisaeta distributa *

Nitrite reductase, Cu-containing	NirK		1 [2?]	(1) *Haloarcula, Halobiforma, Haloferax, Halogeometricum, Halomicrobium, Halopiger, Halorhabdus, Haloterrigena, Natrinema,* and *Natronomonas *

Nitrite reductase, Heme-containing	NirS		1	(1) *Pyrobaculum aerophilum, P. arsenaticum,* and *P. calidifontis *

Nitric oxide reductase	NorB	NarGH in *Pyrobaculum *spp.	4	(1) *Haladaptatus, Haloarcula, Halobiforma, Haloferax, Halogeometricum, Halomicrobium, Halopiger, Halorubrum, Haloterrigena,* and *Natrinema* (2) *Caldivirga, P.* spp., *Vulcanisaeta distributa,* and *Thermoproteus uzoniensis* (3) *Acidilobus* (4) *S. islandicus* spp., *S. solfataricus *spp.

Nitrous oxide reductase	NosZ		3	(1) *Haloarcula, Halobacterium, Halobiforma, Haloferax, Halogeometricum, Halopiger,* and *Halorubrum* (2) *Ferroglobus* (3) *P. calidifontis, P.* sp. 1860

Nitrogenase reductase	NifH	NifD and NifE in Methanococcales, Methanobacteriales, Methanomicrobiales, and *Methanosarcina *	4	(1) Methanococcales (cluster II) (2) Methanobacteriales (cluster II) (3) Methanomicrobiales Methanosarcinales (cluster II) (4) *Methanosarcina* (cluster III)

Nitrogenase, alpha subunit	NifD	NifH and NifE in Methanococcales, Methanobacteriales, Methanomicrobiales, and *Methanosarcina *	(4)	(1) Methanococcales (cluster II) (2) Methanobacteriales (cluster II) (3) Methanomicrobiales Methanosarcinales (cluster II) (4) *Methanosarcina* (cluster III)

Nitrogenase Fe Mo cofactor	NifE	Nif H and NifD in Methanococcales, Methanobacteriales, Methanomicrobiales, and *Methanosarcina *	(4)	(1) Methanococcales (cluster II) (2) Methanobacteriales (cluster II) (3) Methanomicrobiales *Methanosarcina* (cluster II) (4) *Methanosarcina* (cluster III)

**Table 4:** Organic carbon degradation.

Gene name	Gene	Linked to	Number of LGT events	Monophyletic clades
Chitinase and chitin degradation	ChiA	GlmA, GlmD, and Dak in Thermococcales	2	(1) Thermococcales (2) Halobacteriales

Phenol/toluene/xylene hydroxylases		*S. islandicus-S. solfataricus *catechol dioxygenase, *P. arsenaticum *class III extradiol dioxygenases	(2)	(1) *S. islandicus, S. Solfataricus* (2) *P. arsenaticum *

Hydroxyphenylacetate hydroxylases	HpaH	Sulfolobales HpaD	(1)	(1) Sulfolobales (2) *P. oguniense *

Class II extradiol dioxygenases	HpaD	Sulfolobales HpaH, *S. islandicus-S. solfataricus *class III extradiol dioxygenases; *S. islandicus-S. solfataricus* phenol/toluene hydroxylases	3	(1) Sulfolobales (HpaH and catechol dioxygenase) (2) *P. oguniense *

Class III extradiol dioxygenases		*S. islandicus* HpaD, *P. arsenaticum *phenol/toluene hydroxylases, and Sulfolobales hydroxyphenylacetate hydroxylases	6	(1) Sulfolobales (2) *Tp. Acidophilum* (3) *P. arsenaticum, P. Aerophilum* (4) *Methanobacterium* sp. AL-21, SWAN (5) *Methanosphaera* and *Methanoregula* (6) *Methanosarcina acetivorans*, *M. barkeri *

**Table 5:** Calculated lateral transfer rates across the domain Archaea.

Genes	Number of LGT events	LGT rate (events per billion years)*
Cytochrome oxidase	12–14	3.4–4.0
Quinol oxidase	8	2.3
Total genes using oxygen as terminal electron acceptor	**20–22**	**5.7–6.3**

Dissimilatory sulfite reductase (DsrAB)	2	0.6
Thiosulfate sulfurtransferase (SseA)	11	3.1
Sulfur oxygenase reductase (SOR)	2	0.6
Sulfur reductase (SreC)	1	0.3
Flavocytochrome c sulfide dehydrogenase (FCDS)	3	0.9
Sulfide quinone oxidoreductase (SQO)	2	0.6
Total genes using sulfur compounds as electron donors or acceptors	**21**	**6.0**

Nitrate reductase (NarGH)	7	2.0
Nitrite reductase (NirK)	1	0.3
Nitrite reductase (NirS)	1	0.3
Nitric oxide reductase (NorB)	4	1.1
Nitrous oxide reductase (NosZ)	3	0.9
Nitrogenase (NifHDE)	4	1.1
Total genes involved in nitrate reduction or nitrogen fixation	**20**	**5.7**

Chitin degradation	2	0.6
Oxidation of phenolic compounds	9	2.6
Total selected organic carbon degradation genes	**11**	**3.1**

*Assuming the archaeal domain of life is 3.5 billion years old [10].

## References

[1] Gribaldo S, Brochier C (2009). Phylogeny of prokaryotes: does it exist and why should we care?. *Research in Microbiology*.

[2] Abby SS, Tannier E, Gouy M, Daubin V (2012). Lateral gene transfer as a support for the tree of life. *Proceedings of the National Academy of Sciences of the United States of America*.

[3] Blank CE (2009). Phylogenomic dating—the relative antiquity of archaeal metabolic and physiological traits. *Astrobiology*.

[4] Jain R, Rivera MC, Lake JA (1999). Horizontal gene transfer among genomes: the complexity hypothesis. *Proceedings of the National Academy of Sciences of the United States of America*.

[5] Pál C, Papp B, Lercher MJ (2005). Adaptive evolution of bacterial metabolic networks by horizontal gene transfer. *Nature Genetics*.

[6] Comas I, Moya A, González-Candelas F (2007). Phylogenetic signal and functional categories in Proteobacteria genomes. *BMC Evolutionary Biology*.

[7] Popa O, Hazkani-Covo E, Landan G, Martin W, Dagan T (2011). Directed networks reveal genomic barriers and DNA repair bypasses to lateral gene transfer among prokaryotes. *Genome Research*.

[8] Dagan T, Artzy-Randrup Y, Martin W (2008). Modular networks and cumulative impact of lateral transfer in prokaryote genome evolution. *Proceedings of the National Academy of Sciences of the United States of America*.

[9] Wagner A (2009). Evolutionary constraints permeate large metabolic networks. *BMC Evolutionary Biology*.

[10] Blank CE (2009). Not so old Archaea—the antiquity of biogeochemical processes in the archaeal domain of life. *Geobiology*.

[11] Blank CE (2011). An expansion of age constraints for microbial clades that lack a conventional fossil record using phylogenomic dating. *Journal of Molecular Evolution*.

[12] Letsch HO, Kück P, Stocsits RR, Misof B (2010). The impact of rRNA secondary structure consideration in alignment and tree reconstruction: simulated data and a case study on the phylogeny of hexapods. *Molecular Biology and Evolution*.

[13] Thompson JD, Gibson TJ, Higgins DG (2002). Multiple sequence alignment using ClustalW and ClustalX. *Current Protocols in Bioinformatics*.

[14] Rambaut A http://tree.bio.ed.ac.uk/software/seal/.

[15] Ronquist F, Teslenko M, Van Der Mark P (2012). Mrbayes 3.2: efficient bayesian phylogenetic inference and model choice across a large model space. *Systematic Biology*.

[16] Swofford D (2001). *PAUP*. Phylogenetic Analysis Using Parsimony (*and Other Methods), V. 4.0b10*.

[17] Sanderson MJ (2002). Estimating absolute rates of molecular evolution and divergence times: a penalized likelihood approach. *Molecular Biology and Evolution*.

[18] Sanderson MJ (2003). r8s: inferring absolute rates of molecular evolution and divergence times in the absence of a molecular clock. *Bioinformatics*.

[19] Blank CE (2009). Phylogenomic dating—a method of constraining the age of microbial taxa that lack a conventional fossil record. *Astrobiology*.

[20] Maddison WP, Maddison DR http://www.mesquiteproject.org/.

[21] Dutilh BE, van Noort V, van der Heijden RTJM, Boekhout T, Snel B, Huynen MA (2007). Assessment of phylogenomic and orthology approaches for phylogenetic inference. *Bioinformatics*.

[22] Shimodaira H, Hasegawa M (2002). CONSEL: for assessing the confidence of phylogenetic tree selection. *Bioinformatics*.

[23] Sousa FL, Alves RJ, Ribeiro MA, Pereira-Leal JB, Teixeira M, Pereira MM (2012). The superfamily of heme-copper oxygen reductases: types and evolutionary considerations. *Biochimica et Biophysica Acta*.

[24] Brochier-Armanet C, Talla E, Gribaldo S (2009). The multiple evolutionary histories of dioxygen reductases: implications for the origin and evolution of aerobic respiration. *Molecular Biology and Evolution*.

[25] Kozubal MA, Dlakić M, Macur RE, Inskeep WP (2011). Terminal oxidase diversity and function in “*Metallosphaera yellowstonensis*”: gene expression and protein modeling suggest mechanisms of Fe(II) oxidation in the *Sulfolobales*. *Applied and Environmental Microbiology*.

[26] Hipp WM, Pott AS, Thum-Schmitz N, Faath I, Dahl C, Trüper HG (1997). Towards the phylogeny of APS reductases and sirohaem sulfite reductases in sulfate-reducing and sulfur-oxidizing prokaryotes. *Microbiology*.

[27] Acosta M, Beard S, Ponce J, Vera M, Mobarec JC, Jerez CA (2005). Identification of putative sulfurtransferase genes in the extremophilic *Acidithiobacillus ferrooxidans* ATCC 23270 genome: structural and functional characterization of the proteins. *OMICS A Journal of Integrative Biology*.

[28] Xu XW, Wu YH, Zhang HB, Wu M (2007). *Halorubrum arcis* sp. nov., an extremely halophilic archaeon isolated from a saline lake on the Qinghai-Tibet Plateau, China. *International Journal of Systematic and Evolutionary Microbiology*.

[29] Falb M, Müller K, Königsmaier L (2008). Metabolism of halophilic archaea. *Extremophiles*.

[30] Sako Y, Nomura N, Uchida A (1996). *Aeropyrum pernix* gen. nov., sp. nov., a novel aerobic hyperthermophilic archaeon growing at temperatures up to 100°C. *International Journal of Systematic Bacteriology*.

[31] Sako Y, Nunoura T, Uchida A (2001). *Pyrobaculum oguniense* sp. nov., a novel facultatively aerobic and hyperthermophilic archaeon growing at up to 97°C. *International Journal of Systematic and Evolutionary Microbiology*.

[32] Boone & Castenholz (2001). *Bergey’s Manual of Systematic Bacteriology*.

[33] Itoh T, Suzuki KI, Sanchez PC, Nakase T (1999). *Caldivirga maquilingensis* gen. nov., sp. nov., a new genus of rod- shaped crenarchaeote isolated from a hot spring in the Philippines. *International Journal of Systematic Bacteriology*.

[34] Itoh T, Suzuki KI, Nakase T (2002). *Vulcanisaeta distributa* gen. nov., sp. nov., and *Vulcanisaeta souniana* sp. nov., novel hyperthermophilic, rod-shaped crenarchaeotes isolated from hot springs in Japan. *International Journal of Systematic and Evolutionary Microbiology*.

[35] Chen Z, Jiang C, Liu S (2009). Site-directed mutagenesis reveals new and essential elements for iron-coordination of the sulfur oxygenase reductase from the acidothermophilic *Acidianus tengchongensis*. *Chinese Science Bulletin*.

[36] Laska S, Lottspeich F, Kletzin A (2003). Membrane-bound hydrogenase and sulfur reductase of the hyperthermophilic and acidophilic archaeon *Acidianus ambivalens*. *Microbiology*.

[37] Martínez-Espinosa RM, Richardson DJ, Butt JN, Bonete MJ (2006). Respiratory nitrate and nitrite pathway in the denitrifier haloarchaeon *Haloferax mediterranei*. *Biochemical Society Transactions*.

[38] Staples CR, Lahiri S, Raymond J, Von Herbulis L, Mukhophadhyay B, Blankenship RE (2007). Expression and association of group IV nitrogenase NifD and NifH homologs in the non-nitrogen-fixing archaeon *Methanocaldococcus jannaschii*. *Journal of Bacteriology*.

[39] Kendall MM, Liu Y, Sieprawska-Lupa M, Stetter KO, Whitman WB, Boone DR (2006). Methanococcus aeolicus sp. nov., a mesophilic, *Methanogenic archaeon* from shallow and deep marine sediments. *International Journal of Systematic and Evolutionary Microbiology*.

[40] Mehta MP, Baross JA (2006). Nitrogen fixation at 92°C by a hydrothermal vent archaeon. *Science*.

[41] Raymond J, Siefert JL, Staples CR, Blankenship RE (2004). The Natural History of Nitrogen Fixation. *Molecular Biology and Evolution*.

[42] Fani R, Gallo R, Liò P (2000). Molecular evolution of nitrogen fixation: the evolutionary history of the *nif*D, *nif*K, *nif*E, and *nif*N genes. *Journal of Molecular Evolution*.

[43] Zillig W, Boone DR, Catenholz RW (2001). IV. Thermococci. *Bergey’s Manual of Systematic Bacteriology*.

[44] Teske A, Edgcomb V, Rivers AR (2009). A molecular and physiological survey of a diverse collection of hydrothermal vent *Thermococcus* and *Pyrococcus* isolates. *Extremophiles*.

[45] Imanaka T, Fukui T, Fujiwara S (2001). Chitinase from *Thermococcus kodakaraensis* KOD1. *Methods in Enzymology*.

[46] Gao J, Bauer MW, Shockley KR, Pysz MA, Kelly RM (2003). Growth of hyperthermophilic archaeon *Pyrococcus furiosus* on chitin involves two family 18 chitinases. *Applied and Environmental Microbiology*.

[47] Tanaka T, Fukui T, Atomi H, Imanaka T (2003). Characterization of an exo-*β*-D-glucosaminidase involved in a novel chitinolytic pathway from the hyperthermophilic archaeon *Thermococcus kodakaraensis* KOD1. *Journal of Bacteriology*.

[48] Tanaka T, Fukui T, Fujiwara S, Atomi H, Imanaka T (2004). Concerted action of diacetylchitobiose deacetylase and exo-*β*-D- glucosaminidase in a novel chitinolytic pathway in the hyperthermophilic archaeon *Thermococcus kodakaraensis* KOD1. *The Journal of Biological Chemistry*.

[49] Yoshinobu H, Motosuke S, Keita O (2006). Characterization of recombinant family 18 chitinase from extremely halophilic archaeon *halobacterium salinarum* strain NRC-1. *Chitin and Chitosan Research*.

[50] Izzo V, Notomista E, Picardi A, Pennacchio F, Di Donato A (2005). The thermophilic archaeon *Sulfolobus solfataricus* is able to grow on phenol. *Research in Microbiology*.

[51] Chae JC, Kim E, Bini E, Zylstra GJ (2007). Comparative analysis of the catechol 2,3-dioxygenase gene locus in thermoacidophilic archaeon *Sulfolobus solfataricus* strain 98/2. *Biochemical and Biophysical Research Communications*.

